# Supplementary material for: A Critical View of the Application of the APEX Software (Aqueous Photochemistry of Environmentally-Occurring Xenobiotics) to Predict Photoreaction Kinetics in Surface Freshwaters
Source: Molecules. 2019 Dec 18;25(1):9. doi: 10.3390/molecules25010009 (PMC7017383; doi:10.3390/molecules25010009)
Supplement: Supplementary file 1 [file molecules-25-00009-s001.zip › 3.2.4_gcc-4.4.0.zip!/3.2.4_gcc-4.4.0/3.2.4_gcc-4.4.0/doc/octave/html/interpreter/Iterative-Techniques.html]

Iterative Techniques - Untitled


Next: Real Life Example,
Previous: Sparse Linear Algebra,
Up: Sparse Matrices


---

### 21.3 Iterative Techniques applied to sparse matrices

The left division `\` and right division `/` operators,
discussed in the previous section, use direct solvers to resolve a
linear equation of the form x `=` A `\` b or
x `=` b `/` A. Octave equally includes a number of
functions to solve sparse linear equations using iterative techniques.

— Function File: x = **pcg** (a, b, tol, maxit, m1, m2, x0, ...)  
— Function File: [x, flag, relres, iter, resvec, eigest] = **pcg** (...)  
> Solves the linear system of equations a `*` x `=` b by means of the Preconditioned Conjugate Gradient iterative
> method. The input arguments are
>
> - a can be either a square (preferably sparse) matrix or a
>   function handle, inline function or string containing the name
>   of a function which computes a `*` x. In principle
>   a should be symmetric and positive definite; if `pcg`
>   finds a to not be positive definite, you will get a warning
>   message and the flag output parameter will be set.- b is the right hand side vector.- tol is the required relative tolerance for the residual error,
>       b `-` a `*` x. The iteration stops if `norm
>       (`b `-` a `*` x`) <=` tol `* norm (`b `-` a `*` x0`)`. If tol is empty or is omitted, the function sets
>       tol `= 1e-6` by default.- maxit is the maximum allowable number of iterations; if
>         `[]` is supplied for `maxit`, or `pcg` has less
>         arguments, a default value equal to 20 is used.- m = m1 \* m2 is the (left) preconditioning matrix, so that the iteration is
>           (theoretically) equivalent to solving by `pcg` P `*` x `=` m `\` b, with P `=` m `\` a.
>           Note that a proper choice of the preconditioner may dramatically
>           improve the overall performance of the method. Instead of matrices
>           m1 and m2, the user may pass two functions which return
>           the results of applying the inverse of m1 and m2 to
>           a vector (usually this is the preferred way of using the preconditioner).
>           If `[]` is supplied for m1, or m1 is omitted, no
>           preconditioning is applied. If m2 is omitted, m = m1
>           will be used as preconditioner.- x0 is the initial guess. If x0 is empty or omitted, the
>             function sets x0 to a zero vector by default.
>
> The arguments which follow x0 are treated as parameters, and
> passed in a proper way to any of the functions (a or m)
> which are passed to `pcg`. See the examples below for further
> details. The output arguments are
>
> - x is the computed approximation to the solution of
>   a `*` x `=` b.- flag reports on the convergence. flag `= 0` means
>     the solution converged and the tolerance criterion given by tol
>     is satisfied. flag `= 1` means that the maxit limit
>     for the iteration count was reached. flag `= 3` reports that
>     the (preconditioned) matrix was found not positive definite.- relres is the ratio of the final residual to its initial value,
>       measured in the Euclidean norm.- iter is the actual number of iterations performed.- resvec describes the convergence history of the method.
>           resvec `(i,1)` is the Euclidean norm of the residual, and
>           resvec `(i,2)` is the preconditioned residual norm,
>           after the (i-1)-th iteration, i `=
>           1, 2, ...,` iter`+1`. The preconditioned residual norm
>           is defined as
>           `norm (`r`) ^ 2 =` r`' * (`m `\` r`)` where
>           r `=` b `-` a `*` x, see also the
>           description of m. If eigest is not required, only
>           resvec `(:,1)` is returned.- eigest returns the estimate for the smallest eigest `(1)` and largest eigest `(2)` eigenvalues of the
>             preconditioned matrix P `=` m `\` a. In
>             particular, if no preconditioning is used, the estimates for the
>             extreme eigenvalues of a are returned. eigest `(1)`
>             is an overestimate and eigest `(2)` is an underestimate,
>             so that eigest `(2) /` eigest `(1)` is a lower bound
>             for `cond (`P`, 2)`, which nevertheless in the limit should
>             theoretically be equal to the actual value of the condition number.
>             The method which computes eigest works only for symmetric positive
>             definite a and m, and the user is responsible for
>             verifying this assumption.
>
> Let us consider a trivial problem with a diagonal matrix (we exploit the
> sparsity of A)
>
> ```
>           	n = 10;
>           	a = diag (sparse (1:n));
>           	b = rand (n, 1);
>                [l, u, p, q] = luinc (a, 1.e-3);
> ```
>
> Example 1: Simplest use of `pcg`
>
> ```
>             x = pcg(A,b)
> ```
>
> Example 2: `pcg` with a function which computes
> a `*` x
>
> ```
>             function y = apply_a (x)
>               y = [1:N]'.*x;
>             endfunction
>           
>             x = pcg ("apply_a", b)
> ```
>
> Example 3: `pcg` with a preconditioner: l \* u
>
> ```
>           x = pcg (a, b, 1.e-6, 500, l*u);
> ```
>
> Example 4: `pcg` with a preconditioner: l \* u.
> Faster than Example 3 since lower and upper triangular matrices
> are easier to invert
>
> ```
>           x = pcg (a, b, 1.e-6, 500, l, u);
> ```
>
> Example 5: Preconditioned iteration, with full diagnostics. The
> preconditioner (quite strange, because even the original matrix
> a is trivial) is defined as a function
>
> ```
>             function y = apply_m (x)
>               k = floor (length (x) - 2);
>               y = x;
>               y(1:k) = x(1:k)./[1:k]';
>             endfunction
>           
>             [x, flag, relres, iter, resvec, eigest] = ...
>                                pcg (a, b, [], [], "apply_m");
>             semilogy (1:iter+1, resvec);
> ```
>
> Example 6: Finally, a preconditioner which depends on a
> parameter k.
>
> ```
>             function y = apply_M (x, varargin)
>             K = varargin{1};
>             y = x;
>             y(1:K) = x(1:K)./[1:K]';
>             endfunction
>           
>             [x, flag, relres, iter, resvec, eigest] = ...
>                  pcg (A, b, [], [], "apply_m", [], [], 3)
> ```
>
> References
>
> [1] C.T.Kelley, 'Iterative methods for linear and nonlinear equations',
> SIAM, 1995 (the base PCG algorithm)
>
> [2] Y.Saad, 'Iterative methods for sparse linear systems', PWS 1996
> (condition number estimate from PCG) Revised version of this book is
> available online at http://www-users.cs.umn.edu/~saad/books.html
>
>
>
> **See also:** sparse, pcr.

— Function File: x = **pcr** (a, b, tol, maxit, m, x0, ...)  
— Function File: [x, flag, relres, iter, resvec] = **pcr** (...)  
> Solves the linear system of equations a `*` x `=` b by means of the Preconditioned Conjugate Residuals iterative
> method. The input arguments are
>
> - a can be either a square (preferably sparse) matrix or a
>   function handle, inline function or string containing the name
>   of a function which computes a `*` x. In principle
>   a should be symmetric and non-singular; if `pcr`
>   finds a to be numerically singular, you will get a warning
>   message and the flag output parameter will be set.- b is the right hand side vector.- tol is the required relative tolerance for the residual error,
>       b `-` a `*` x. The iteration stops if `norm
>       (`b `-` a `*` x`) <=` tol `* norm (`b `-` a `*` x0`)`. If tol is empty or is omitted, the function sets
>       tol `= 1e-6` by default.- maxit is the maximum allowable number of iterations; if
>         `[]` is supplied for `maxit`, or `pcr` has less
>         arguments, a default value equal to 20 is used.- m is the (left) preconditioning matrix, so that the iteration is
>           (theoretically) equivalent to solving by `pcr` P `*` x `=` m `\` b, with P `=` m `\` a.
>           Note that a proper choice of the preconditioner may dramatically
>           improve the overall performance of the method. Instead of matrix
>           m, the user may pass a function which returns the results of
>           applying the inverse of m to a vector (usually this is the
>           preferred way of using the preconditioner). If `[]` is supplied
>           for m, or m is omitted, no preconditioning is applied.- x0 is the initial guess. If x0 is empty or omitted, the
>             function sets x0 to a zero vector by default.
>
> The arguments which follow x0 are treated as parameters, and
> passed in a proper way to any of the functions (a or m)
> which are passed to `pcr`. See the examples below for further
> details. The output arguments are
>
> - x is the computed approximation to the solution of
>   a `*` x `=` b.- flag reports on the convergence. flag `= 0` means
>     the solution converged and the tolerance criterion given by tol
>     is satisfied. flag `= 1` means that the maxit limit
>     for the iteration count was reached. flag `= 3` reports t
>     `pcr` breakdown, see [1] for details.- relres is the ratio of the final residual to its initial value,
>       measured in the Euclidean norm.- iter is the actual number of iterations performed.- resvec describes the convergence history of the method,
>           so that resvec `(i)` contains the Euclidean norms of the
>           residual after the (i-1)-th iteration, i `=
>           1,2, ...,` iter`+1`.
>
> Let us consider a trivial problem with a diagonal matrix (we exploit the
> sparsity of A)
>
> ```
>           	n = 10;
>           	a = sparse (diag (1:n));
>           	b = rand (N, 1);
> ```
>
> Example 1: Simplest use of `pcr`
>
> ```
>             x = pcr(A, b)
> ```
>
> Example 2: `pcr` with a function which computes
> a `*` x.
>
> ```
>             function y = apply_a (x)
>               y = [1:10]'.*x;
>             endfunction
>           
>             x = pcr ("apply_a", b)
> ```
>
> Example 3: Preconditioned iteration, with full diagnostics. The
> preconditioner (quite strange, because even the original matrix
> a is trivial) is defined as a function
>
> ```
>             function y = apply_m (x)
>               k = floor (length(x)-2);
>               y = x;
>               y(1:k) = x(1:k)./[1:k]';
>             endfunction
>           
>             [x, flag, relres, iter, resvec] = ...
>                                pcr (a, b, [], [], "apply_m")
>             semilogy([1:iter+1], resvec);
> ```
>
> Example 4: Finally, a preconditioner which depends on a
> parameter k.
>
> ```
>             function y = apply_m (x, varargin)
>               k = varargin{1};
>               y = x; y(1:k) = x(1:k)./[1:k]';
>             endfunction
>           
>             [x, flag, relres, iter, resvec] = ...
>                                pcr (a, b, [], [], "apply_m"', [], 3)
> ```
>
> References
>
> [1] W. Hackbusch, "Iterative Solution of Large Sparse Systems of
> Equations", section 9.5.4; Springer, 1994
>
>
>
> **See also:** sparse, pcg.

The speed with which an iterative solver converges to a solution can be
accelerated with the use of a pre-conditioning matrix M. In this
case the linear equation M`^-1 *` x `=` M`^-1 *` A `\` b is solved instead. Typical pre-conditioning matrices
are partial factorizations of the original matrix.

— Loadable Function: [l, u, p, q] = **luinc** (a, '0')  
— Loadable Function: [l, u, p, q] = **luinc** (a, droptol)  
— Loadable Function: [l, u, p, q] = **luinc** (a, opts)  
> Produce the incomplete LU factorization of the sparse matrix a.
> Two types of incomplete factorization are possible, and the type
> is determined by the second argument to luinc.
>
> Called with a second argument of '0', the zero-level incomplete
> LU factorization is produced. This creates a factorization of a
> where the position of the non-zero arguments correspond to the same
> positions as in the matrix a.
>
> Alternatively, the fill-in of the incomplete LU factorization can
> be controlled through the variable droptol or the structure
> opts. The UMFPACK multifrontal factorization code by Tim A.
> Davis is used for the incomplete LU factorization, (availability
> http://www.cise.ufl.edu/research/sparse/umfpack/)
>
> droptol determines the values below which the values in the LU
> factorization are dropped and replaced by zero. It must be a positive
> scalar, and any values in the factorization whose absolute value are
> less than this value are dropped, expect if leaving them increase the
> sparsity of the matrix. Setting droptol to zero results in a
> complete LU factorization which is the default.
>
> opts is a structure containing one or more of the fields
>
> `droptol`: The drop tolerance as above. If opts only contains `droptol` then this is equivalent to using the variable droptol. `milu`: A logical variable flagging whether to use the modified incomplete LU factorization. In the case that `milu` is true, the dropped values are subtracted from the diagonal of the matrix U of the factorization. The default is `false`. `udiag`: A logical variable that flags whether zero elements on the diagonal of U should be replaced with droptol to attempt to avoid singular factors. The default is `false`. `thresh`: Defines the pivot threshold in the interval [0,1]. Values outside that range are ignored.
>
> All other fields in opts are ignored. The outputs from luinc
> are the same as for lu.
>
> Given the string argument 'vector', luinc returns the values of p
> q as vector values.
>
>
>
> **See also:** sparse, lu.
